# Operational and data-integrity limitations of a system-wide Epic OPAT order set: a three-year cohort analysis

**DOI:** 10.1017/ash.2026.10402

**Published:** 2026-05-21

**Authors:** M. Yasser Alsafadi, Shivani Patel, Natalie Finch, Shemual Tsai, Wesley Hoffmann

**Affiliations:** 1 Division of Infectious Diseases, Houston Methodist Hospital, Houston, TX, USA; 2 Department of Pharmacy, Houston Methodist Hospital, Houston, TX, USA

## Abstract

We evaluated an Epic-based outpatient parenteral antimicrobial therapy (OPAT) order set to assess its impact on clinical workflows and analytics. Free text, missing structured fields, and lack of microbiology linkage made it difficult to track care, monitor safety, and analyze outcomes, highlighting essential design requirements for OPAT system rebuilds.

## Introduction

Outpatient parenteral antimicrobial therapy (OPAT) programs enable patients to receive intravenous antimicrobials after hospital discharge in outpatient settings. High-quality OPAT care relies on accurate data capture, reliable transitions of care, and structured clinical workflows. Within our health system, we identified safety concerns, including missed therapeutic drug monitoring (TDM), unclear regimen instructions, and variable transitions across infusion sites. This motivated a system-level evaluation of our Epic-based OPAT order set and the data architecture supporting OPAT care. Prior studies have shown that 30-day readmissions among OPAT patients are common and associated with both clinical and system-level factors, underscoring the importance of reliable OPAT infrastructure and monitoring processes.^1^ As OPAT programs increasingly depend on electronic health records for care coordination and surveillance, deficiencies in Electronic Health Record design can directly shape clinical workflows, data integrity, and quality control.^2^

In response to an elevated 30-day revisit rate, we conducted a detailed evaluation to understand how our current custom OPAT build might influence clinical operations and analytic performance. Our objectives were to (1) identify key operational and data-model limitations within the current OPAT build, (2) evaluate their impact on analytic reproducibility and clinical workflows, and (3) define essential elements to inform a future system rebuild. Table 1 summarizes the characteristics of OPAT episodes across a three-year health system cohort.

**Table 1.** Characteristics of OPAT episodes (N-9,490)

Characteristic	Value
Age, years, median (Interquartile Range)	64 (51–73)
Gender	
Female	4,375 (46.1%)
Male	5,115 (53.9%)
ID involvement	9,015 (95.0%)
Surgical case	3,445 (36.3%)
**OPAT indication**	
Central nervous system infection	52 (0.5%)
Intra-abdominal infection	217 (2.3%)
Respiratory infection	245 (2.6%)
Bone and joint infection	391 (4.1%)
Urinary tract infection	589 (6.2%)
Endovascular infection	940 (9.9%)
Other	1,171 (12.3%)
Skin and soft tissue infection	1,289 (13.6%)
Sepsis (unknown source)	4,596 (48.4%)
**Antibiotic class (selected categories)**	
Vancomycin	974 (10.3%)
Aminoglycoside	51 (0.5%)
Antifungal	310 (3.3%)
Antiviral	246 (2.6%)
XDR-targeted agent	320 (3.4%)
Narrow-spectrum *β*-lactam	722 (7.6%)
Other MRSA-active agent	1,631 (17.2%)
**Outcomes**	
30-day inpatient readmission	1,773 (18.7%)
All-cause 30-day revisit	2,986 (31.5%)

Values represent OPAT episodes occurring between January 3, 2022, and February 28, 2025; individual patients may contribute more than one episode. Diagnosis category was derived from International Classification of Diseases (ICD-10) codes and may not reflect the true OPAT indication. Antibiotic class variables represent selected analytic categories and do not encompass all OPAT regimens.

### Setting and workflow description

At our institution, OPAT orders are placed through a single Epic order set that is embedded within a case management consult, rather than being integrated into core clinical workflows such as admission, progress note documentation, or discharge planning. The order set is a custom build that relies heavily on unstructured free-text fields for key elements, including antibiotic name, dose, frequency, and TDM instructions. Discrete data fields are not available for OPAT indication, identified causative organism or antimicrobial susceptibility data, infusion location, line type, responsible outpatient provider, or total intended therapy duration. In its current configuration, the order set does not generate an OPAT episode of care, populate an OPAT registry, or interface directly with microbiology systems.

Reconstructing OPAT episodes, therefore, required manual linkage of data across multiple sources, including:

- Epic (orders, encounters).
- ICD-10 billing data (used as a surrogate for indication).
- Microbiology platforms (which were too noisy for reliable structured linkage).

Multiple data extraction iterations were required due to the absence of standardized mapping tables or OPAT identifiers.

## Results

### Indication patterns reveal structural failures in data capture

Across 9,490 OPAT episodes, occurring between January 3, 2022, and February 28, 2025, Table 1 shows that “Sepsis (unknown source)” accounted for 48.4% of episodes, while “other” indications accounted for 12.3%. In contrast, classic OPAT indications (eg, endovascular, bone/joint, skin and soft tissue infection, intra-abdominal infection) represented only a minority of cases. This distribution reflects the inadequacy of ICD-10 codes as a surrogate for OPAT indication and underscores the need for a dedicated structured OPAT indication field.

### Antibiotic utilization patterns demonstrate fragmented data architecture

The most frequently used OPAT agents were ertapenem (20.1%), ceftriaxone (14.8%), meropenem (8.7%), daptomycin (7.5%), and cefepime (7.2%). However, the antibiotic class variables presented in Table 1 represent only a limited subset of analytic categories (eg, vancomycin exposure or antifungal therapy) and fail to capture the full breadth of OPAT prescribing practices. This gap highlights the limitations of free-text regimen entry and the absence of structured antimicrobial fields capable of supporting comprehensive analysis.

### Thirty-day readmission and revisit rates as descriptive clinical context

The 30-day inpatient readmission rate, defined as inpatient encounters only based on account class, was 18.7%, while the all-cause 30-day revisit rate, including emergency department, observation, and inpatient encounters, was 31.5%, placing these rates at the higher end of published benchmarks and prior OPAT readmission models.^3^ These findings provide clinical context for the importance of reliable structured OPAT data capture, although the present analysis does not establish a direct causal relationship between workflow limitations and postdischarge utilization.

### Manual effort confirms monitoring impracticality

Over 100 hours of manual data cleaning were required to normalize free-text antibiotic entries. Efforts to map ICD-10 codes to OPAT-relevant indications still resulted in over 60% of episodes being categorized as “sepsis” or “other.” Structured data for organism, resistance pattern, infusion location, line type, and total therapy duration were unavailable, and entire analytic domains (eg, microbiology linkage, line classification) had to be abandoned. Under the current build, routine OPAT surveillance is not feasible.

## Discussion

We identified three order set design flaws with potentially significant impact. First, reliance on free-text entry for antibiotic regimens introduces safety risks, increases the burden of order clarification, and rules out reliable analysis. Second, the absence of a structured OPAT indication field necessitates reliance on ICD-10 codes as a surrogate, resulting in largely uninformative, non-actionable diagnostic categories. Third, the lack of microbiology linkage limits evaluation of treatment adequacy, resistance-related risk, and core antimicrobial stewardship questions.

Collectively, these deficiencies likely impair clinical care delivery, transitions of care, case management operations, and analytic functions. The inability to stratify risk, reliably monitor TDM, or compare OPAT outcomes to published standards exemplifies how data-model limitations translate directly into patient-level and system-level vulnerabilities. For example, prior OPAT cohorts have shown higher readmission risk among patients receiving vancomycin or aminoglycosides and those treated for infections involving prosthetic material, while also identifying frequent gaps in TDM—risk factors that require structured and analyzable OPAT data to identify and address.^4^

Two lessons for other hospitals are clear: 1) core OPAT data should not be captured using free-text fields; and 2) ICD-10 codes cannot substitute for structured OPAT indication data. Prior work has demonstrated that targeted Epic modifications—including structured OPAT order elements and registries—can substantially improve OPAT management and monitoring, supporting the feasibility of system redesign efforts.^5^

### Recommendations

#### For EHR vendors

Develop OPAT-ready modules that incorporate structured fields for indication, antimicrobial agent, dose, frequency, duration, and infusion location; embedded TDM logic; optional microbiology linkage; and automatic OPAT episode creation with patient census lists.

#### For health systems

Customize OPAT indication lists; define monitoring protocols; standardize responsibilities across ID, case management, pharmacy, and home health teams; and ensure consistent use of the OPAT order sets through governance and training.

### Take-home message

Structured capture at the point of entry is an important component of high-quality and safe OPAT care. When critical OPAT information is not recorded in standardized, extractable fields, downstream manual effort may be insufficient to fully overcome those limitations. As a result, clinical workflows, transitions of care, and system-wide analytics may be hindered.
